# Molecular assessment of oyster microbiomes and viromes reveals their potential as pathogen and ecological sentinels

**DOI:** 10.1016/j.onehlt.2025.100973

**Published:** 2025-01-13

**Authors:** Jordan R. Walker, Dennis A. Bente, Megan T. Burch, Filipe M. Cerqueira, Ping Ren, Jessica M. Labonté

**Affiliations:** a Department of Marine Biology, Texas A&M University at Galveston, Galveston 77554, TX, United States; b Department of Microbiology and Immunology, University of Texas Medical Branch, Galveston 77555, TX, United States; c Department of Pathology, University of Texas Medical Branch, Galveston 77555, TX, United States

**Keywords:** Oysters, Microbiome, Food safety, Aquaculture, Viruses

## Abstract

Oyster aquaculture world-wide is a booming industry that can provide many benefits to coastal habitats, including economic, ecosystem-level, and cultural benefits. Oysters present several risks for human consumption, including transmission of parasites, and bacterial and viral pathogens. Oyster microbiomes are well-defined, but their connection to the incidence of pathogens, humans or others, is unclear. Furthermore, viruses associated with oysters are largely unknown, and their connection to humans, animals, and ecosystem health has not been explored. Here, we employed a One Health framework and modern molecular techniques, including 16S rRNA amplicon and metagenomic sequencing, to identify links between changes in the microbial and viral communities associated with oysters and the incidence of pathogens detected in oyster tissues and their surrounding environments. In addition, we adapted the BioFire® FilmArray®, commonly used in hospitals, to determine the presence of human pathogens within the sampled oysters. We detected known human pathogens in 50 % of the oysters tested. Within the genomic datasets, we noted that pathogens of humans, animals, and plants in oysters were shared with the nearby water and sediments, suggesting a sink–source dynamic between the oysters and their surroundings. 16S rRNA gene analysis revealed that while oysters share common microbial constituents with their surrounding environments, they enrich for certain bacteria such as Mycoplasmatales, Fusobacteriales, and Spirochaetales. On the contrary, we found that oyster viromes harbored the same viruses in near equal relative abundances as their surrounding environments. Our results show how oysters could be used not only to determine the risk of human pathogens within coastal estuaries but also how oyster viruses could be used as ecosystem-level sentinels.

## 1 Introduction

Oysters are coastal reef-building organisms that provide substantial ecosystem, monetary, and cultural services to their environment. Oyster reefs provide a plethora of ecological benefits including being a physical barrier protecting shores, the creation of nursery and foraging habitats, and removal of suspended particulate matter [[1], [2], [3], [4], [5]]. Beyond the ecological benefits of oysters, there is historical evidence of oysters being an important food source for human populations worldwide [1,6,7]. Aquaculture of bivalves, including oysters, represents an expanding enterprise with the potential to reintroduce these vital organisms to ecosystems where they have been lost. Commercial oyster landings in the United States were evaluated at 12,747 tons, for an estimated value of $258 million US dollars in 2022 [8]. Furthermore, bivalve aquaculture can increase the coastal ecosystem's cultural services, including wildlife recreational activities, education, and research, which have been estimated to be $6.47 billion US dollars globally [9].

Oysters are ingesting and filtering microbial populations, including bacteria, protists, and viruses, from the surrounding water. To that end, oysters harbor a core microbiome susceptible to transient microbial populations, while still being distinct from the environment [[10], [11], [12], [13], [14]]. Oyster microbiomes provide benefits to their host, such as production of digestive enzymes, defense against pathogens, predator-prey interactions, and adaptation to stress [11,12,[15], [16], [17]]. While bacterial communities within oysters are largely resident, protists and viruses have a more transient relationship [10,14]. Viruses found in oyster tissues are extremely diverse and currently poorly represented in publicly available databases [18].

High contact rates and a propensity to accumulate microorganisms increase the potential for oysters to accumulate human pathogens. The human bacterial pathogens associated with oysters are diverse and include *Vibrio*, *Salmonella*, *Shigella*, *Plesiomonas*, and *Listeria* species, as well as *Escherichia coli* [19,20]. Protozoan parasites are also found in many commercially important shellfish including *Giardia duodenalis*, *Cryptosporidium* spp., and *Toxoplasma gondii* [21]. Viruses that cause disease outbreaks from oyster consumption include Noroviruses, Hepatitis A, Hepatitis E, and poliovirus [19,20]. Oysters near wastewater outflows, which despite treatment still carry pathogens [19,22], are more susceptible to accumulate pathogenic bacteria, protozoans, and viruses. While post-harvest treatment of oysters is employed to limit outbreaks of bacterial diseases, viruses have been shown to persist [19,23]. Additionally, protozoan parasites remain infectious for long periods of time in seawater and shellfish [21].

The recent (2018) legalization of oyster farming in Texas has created a new and expanding source of oysters for human consumption. Oyster mariculture intended for human consumption is a relevant research area for One Health principles, which recognize that the health of people is closely intertwined with the health of animals and their shared environments. Here, we utilized this One Health perspective to explore the potential to utilize oysters as ecosystem and public health sentinels. Oysters have the potential to serve as excellent biological monitors of environmental and human health due to their unique characteristics and interactions with aquatic environments. Oysters act as natural filtration systems, processing water and accumulating various substances from their environment. As filter-feeders, they can reveal critical information about ecosystem health and contamination levels. We aim to explore the potential to use oysters as pathogen sentinels by correlating the detection of human pathogens in oysters and the surrounding environment with changes in the microbial, protozoan, and viral populations. Additionally, we compare the community compositions between oysters and their environments to explore the potential to utilize oysters as sentinels of environmental conditions. We hypothesized that oyster microbiomes and viromes would display similar community composition and functional potential as the surrounding water and sediment, making them suitable as sentinels of pathogens and environmental water quality.

## 2 Materials and methods

### 2.1 Sampling, metadata measurements, and oyster processing

**Fig. 1:**
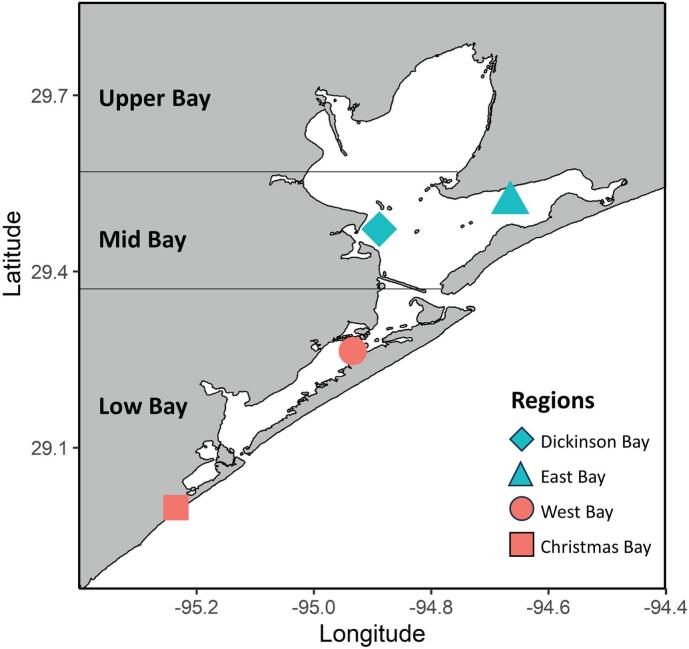
Location of the sampled oyster reefs in Galveston Bay, an estuary in the upper coast of Texas discharging in the western Gulf of Mexico. Blue are subtidal samples where oysters remain submerged throughout tidal fluctuations, and red are intertidal samples where low tides can expose oysters to air. (For interpretation of the references to colour in this figure legend, the reader is referred to the web version of this article.)

Oysters were sampled during October and November 2023 from four separate regions within Galveston Bay: Dickinson Bay, East Bay, West Bay, and Christmas Bay (Fig. 1, Supp. Table 1). Every sampling day began at approximately 8:00 AM. At every station, 5–10 oysters were collected using a mechanical dredge (length 38 cm x width 28 cm x depth 25 cm) towed for no more than 7 min at a speed of 2.5–3 knots, then stored on ice. At Christmas Bay, no living oysters were collected using the mechanical dredge, so they were collected from a nearby dock. At each site, 20 L of water was collected and passed through a Nitex filter. Sediment samples were sampled with a sediment grab and stored on ice until returned to the laboratory. Water temperature, salinity, and dissolved oxygen were recorded using a YSI Professional 2030 instrument (Pro2030). In the lab, sediment samples were stored at 4 °C until all samples were collected and oyster samples were stored at 4 °C no longer than 24 h before processing. Water samples were immediately filtered through 142 mm GFF (0.7 μm) and 0.2 μm PTFE membrane filters then preserved at −80 °C. Viruses in the filtrate were concentrated using a Centramate Tangential Flow Filtration system (Cytiva, Marlborough, MA) with a pore size of 30 kDa to a volume of less than 250 mL. Viral concentrates were stored at 4 °C until further processing.

Oysters were shucked using a flame-sterilized shucking knife and whole oyster tissues were rinsed three times in sterile artificial seawater (0.2 M NaCl, 50 mM Tris–HCl, 5 mM CaCl2, and 5 mM MgCl2, pH 7.5). Oyster slurries were generated by processing whole oyster tissues in a flame-sterilized blender with ∼300 mL of artificial seawater. Ten mL of each slurry was frozen at −80 °C to be used for whole community analysis and pathogen detection. The remaining slurry volume was used for viral particle separation using a modified version of the methods described by Wei et al. [24]. Slurries were frozen at −80 °C and thawed three time, then centrifuged sequentially at 1000, 3000, 5000, 8000, 10,000, and 12,000 ×g at 5 min intervals, retaining the supernatants after each round. The final supernatants were filtered through 42 mm GFD (2.7 μm), GFF (0.7 μm), and 0.2 μm PTFE membrane filters. 10 % *w*/*v* PEG was added to the filtrates to precipitate viral particles. PEG-treated samples were stored at 4 °C overnight and then centrifuged at 13000 ×*g* for 30 min. DNA was immediately extracted from the pellets.

### 2.2 DNA extraction, PCR amplification, and sequencing

DNA was extracted from samples (0.22 μm filters, 2 g sediment, 2 g raw oyster, PEG pellets) using the ZymoBIOMICS DNA/RNA Miniprep Kit per the manufacturer's instructions, except bead beating was skipped for viral samples. We used the generic 16S V4-V5 rRNA region (primers 515F—Y (5’-GTGYCAGCMGCCGCGGTAA) and 926R (5’-CCGYCAATTYMTTTRAGTTT) with Golay barcodes [25,26]) to assess the total bacterial community composition of oysters and their surrounding environment. Amplification was performed in triplicate on the sediment, filters, whole oyster extractions, and a negative (no DNA template) control. Thermocycle conditions were as follows: 3 min at 95 °C; 30 cycles at 95 °C for 45 s, 50 °C for 45 s, and 68 °C for 90 s; 68 °C for 5 min. Triplicates were pooled, and amplification was confirmed using a 2 % agarose gel electrophoresis. PCR products were cleaned using ZymoBIOMICS Select-A-Size DNA Clean & Concentrator to retain DNA fragments above 200 bp, per the manufacturer's instructions. The remainder of DNA extracts (up to 1.7 μg) were sent for DNA sequencing using Illumina NovaSeq 6000 SP with 150 bp paired-end reads. The amplicons were sequenced using Illumina MiSeq with 250 bp paired-end reads. All sequencing was done at the Texas A&M AgriLife Genomics & Bioinformatics Service

### 2.3 BioFire pathogen and *Legionella* spp. identification

In order to determine the presence of medically relevant pathogens we used the BioFire® FilmArray® Gastrointestinal (BF-GI) multiplex PCR kit (bioMerieux, Cambridge, MA) for 22 common GI pathogens (Supp. Table 3) which is a common medical tool using targeted primers for common human pathogens. The aforementioned slurries and 24 additional slurries collected from previous sampling trips, Texas Parks and Wildlife, and the TAMUG Seafood Safety Lab (Supp. Table 2) were tested using the BF-GI. *Legionella* spp. was identified by extracting DNA from oyster slurries using the foodproof® StarPrep Two Kit (BIOTECON Diagnostics) following the *Legionella* instructions for extraction procedure B, without Reagent D and D-light treatment. DNA was analyzed with the microproof® *Legionella* Quantification LyoKit (BIOTECON Diagnostics). qPCR was performed using the LightCycler® 96 Instrument (Roche) and analyzed with the LightCycler® 96 SW 1.1 software (Roche).

### 2.4 Metagenomic analyses

Quality control, including merging of reads, sequencing artifact and adapter trimming, contamination removal, and entropy masking, was performed with the BBTools suite [27]. De novo assembly was completed with MEGAHIT using default parameters and a 1 kbp cutoff [28]. VIBRANT was used predict and confirm which contigs were of viral origin within the metagenomes and viromes [29]. Prodigal was used to predict open reading frames [30]. Protein annotation was accomplished using DIAMOND blastp against the nr database (release 259 downloaded Dec 15, 2023) for the non-virome samples and the RefSeq Viral database (release 222 downloaded January 8, 2024) for the viromes [31]. Data visualization was preformed using MEGAN6 and extracting the summary counts [32].

### 2.5 Microbiome analysis

The amplified 16S rRNA gene sequences were analyzed using the pipeline described by McNichol et al. [33]. Briefly, sequences were split into 16S and 18S rRNA gene files using bbsplit and the SILVA and PR2 databases. The reads were then denoised and annotated using qiime2 and a combination of the SILVA (v. 138.1) and PR2 (v 5.0.0) databases. ASVs related to Metazoans were removed. A 3.2-fold correction was applied for sequencing bias based on the ratio of the molarity of the 16S and 18S peaks identified by smear test. Functional annotations were performed using the standard workflow for Picrust2 and adding the KEGG descriptions [34]. Potential pathogens were predicted by aligning the prokaryotic sequences to the MBPD database using DADA2 [35,36]. It should be noted that the Dickinson Bay oyster sample failed.

### 2.6 Statistical analyses

Relative abundances of ASVs were calculated by normalizing to the total number of sequences per sample and viral genes to the total number of predicted genes in each metagenome. NMDS analysis was performed on centered log-ratio transformed [37] Picrust2 functional relative abundances, viral family gene counts, and AMG gene counts using Euclidean distances on all samples except the ASV counts where Jaccard distance was used due to improved stress values [38]. Environmental parameters were correlated with the NMDS analysis, but no significant correlations were found. PERMANOVA analysis comparing sites and sample type was conducted using the adonis2 function in vegan [38]. Lefse analysis was performed to test which features most likely contributed to the differences in the community or functional profiles using relative abundances of viral genes or amplicons [39]. Lefse results were considered significant if *p* < 0.05 and LDA score > 5.

## 3 Results

### 3.1 Characterization of the oyster and surrounding environment viromes

**Fig. 2:**
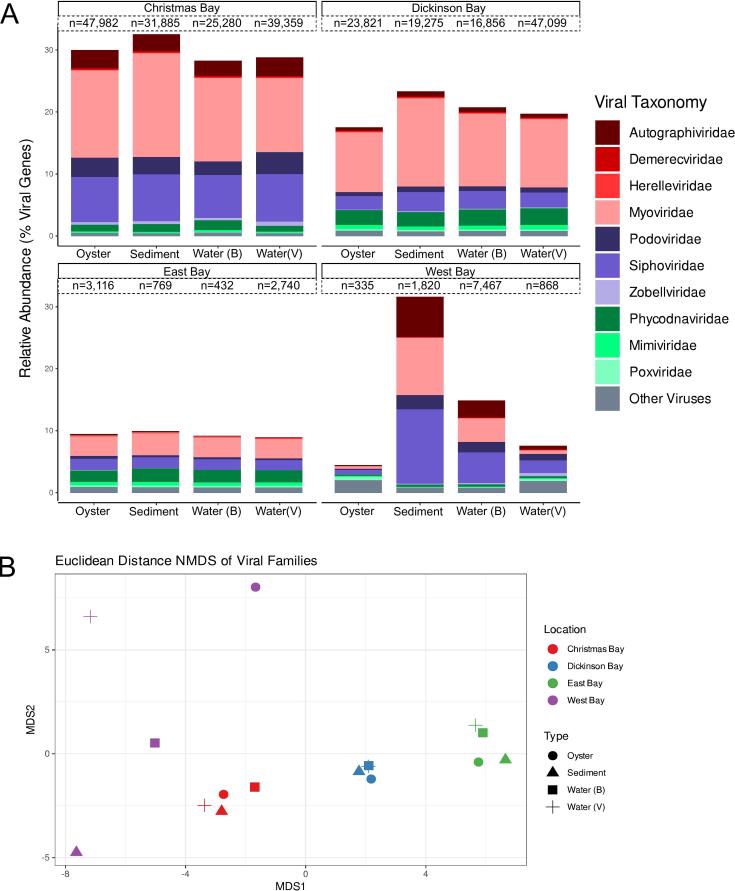
(A) Taxonomic composition of the viromes of oysters and their surrounding water and sediment in four locations in Galveston Bay. The total number of viral contigs identified by VIBRANT is indicated above each bar. The top 10 most abundant viral families within all metagenomes are displayed separately; all other viral families are grouped into “Other Viruses”. (B) NMDS analysis of the family-level taxonomic composition of the viromes of the oysters and surrounding water and sediment.

We used metagenomics to characterize the viral (<0.22 μm) and microbial (total DNA) fractions of the oysters (Supp. Table 4). On average, VIBRANT identified 36,126, 26,763, 2623, and 1764 viral contigs in samples from Christmas Bay, Dickinson Bay, West Bay, and East Bay, respectively (Fig. 2A). Most genes predicted from viral contigs were not represented in the Viral RefSeq database and remain “*unknown*”, with 4.5 to 32 % of the sequences being identified (Fig. 2A). Phages from the cosmopolitan families *Myoviridae* (8 %) and *Podoviridae* (4 %) dominated the identifiable genes of the total genes predicted, with the next most abundant viral families being *Autographiviridae* (1.5 %) and *Phycodnaviridae* (1.5 %) (Fig. 2A). Eukaryotic viruses were mainly algal viruses from the families *Phycodnaviridae* and *Mimiviridae*. *Poxviridae,* which was found ubiquitously, and *Metaviridae,* which was primarily found at West Bay (<0.8 %) and Christmas Bay sediment (0.0005 %) were the most abundant viruses with known Metazoan hosts (Fig. 2A). We observed a high level of similarity between the viromes from the same location but different sample types (Fig. 2). We confirmed the similarity by NMDS analysis of the viral families (stress 0.037) and PERMANOVA grouping by sample location (*p* = 0.001). No viral taxonomic groups met the Lefse thresholds set for significantly contributing to the difference between communities.

### 3.2 Characterization of the microbial communities

**Fig. 3:**
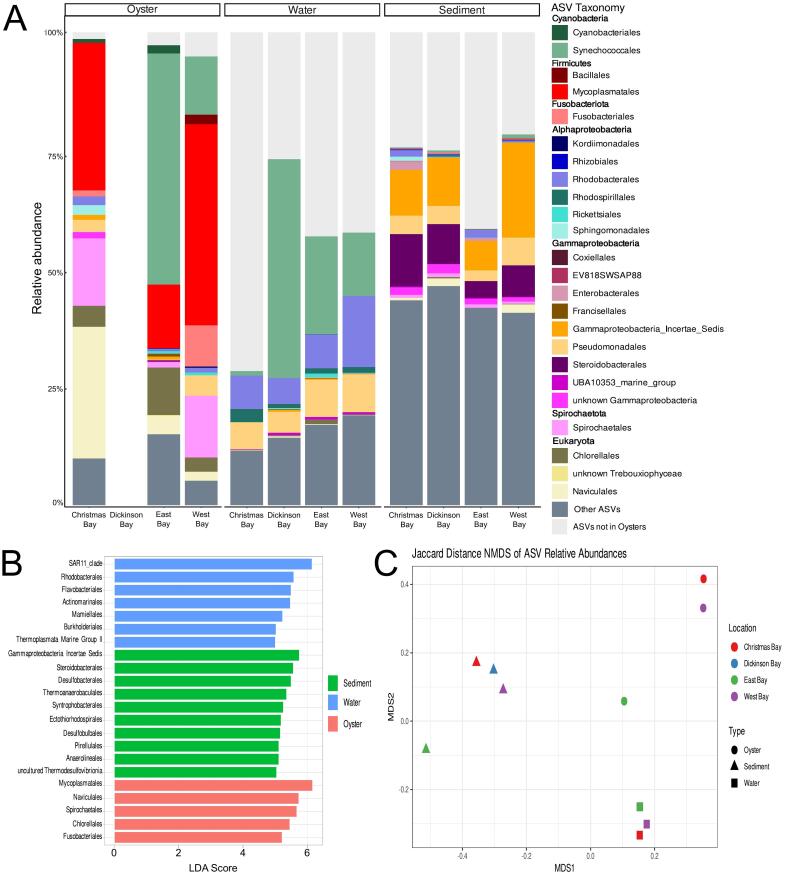
(A) Taxonomic composition of the microbial communities based on 16S rRNA gene sequencing shared between the oyster and their surrounding water and sediment. (B) LefSe analysis results for significantly (α < 0.05) different microbial orders with an LDA score of at least 5 between the three types of samples. (C) NMDS analysis of the presence and absence of ASVs in the samples.

We used rRNA gene sequencing of the V4-V5 regions to assess the bacterial and eukaryotic microbes of the oysters and their surrounding environments. Microbial ASVs found in the oysters and at least one other sample type accounted for at least 95 % of all ASVs found in the oyster microbiomes (Fig. 3A). Although most microbial orders found in the oysters were also found in the other sample types, the relative abundance of the ASVs differed substantially (Fig. 3A-B). The microbial communities of the oysters were enriched in Mycoplasmatales, Fusobacteriales, Spirochaetales, Naviculales, and Chlorellales confirmed by LefSe (Figs. 3A-B). In contrast to the viral communities, the microbial communities were separated by sampling type when analyzed using NMDS analysis and confirmed by PERMANOVA (*p* = 0.001) (Fig. 3C).

### 3.3 Comparison of microbial ecological functions

**Fig. 4:**
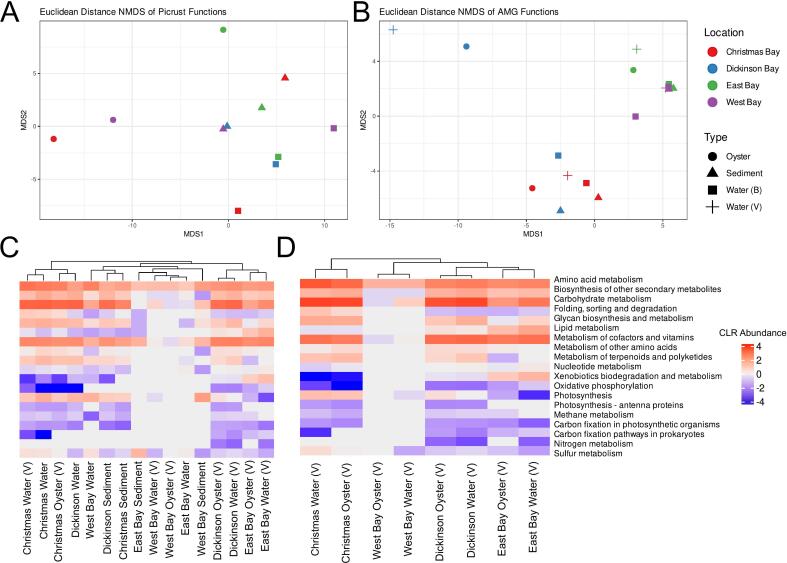
Comparison of the NMDS analysis comparing (A) the KEGG modules identified from the 16S rRNA gene sequence data using Picrust2 and (B) the AMG functional profiles identified by VIBRANT. Hierarchical clustering of the KEGG metabolisms found in AMGs from (C) all samples and (D) excluding the sediment and bacterial water samples. Samples from viromes are indicated by V in parentheses.

We investigated the similarities in the microbial and viral AMGs metabolic function of the samples. LefSe analysis of all functional profiles resulted in no differentially abundant KEGG modules. PERMANOVA analysis was significantly different between sample types (α < 0.05) and the AMGs between sample locations (α < 0.05) (Fig. 4A-B). Hierarchical clustering of the functional profiles of AMGs revealed that the viromes tended to group by sample location, while the AMGs predicted from sediment of bacterial communities of the water column did not show a clear trend (Fig. 4C). Virome grouping based on location was confirmed through hierarchical clustering on AMGs in viral fractions (Fig. 4D).

### 3.4 Identificaiton of known human pathogens in oyster samples

**Fig. 5:**
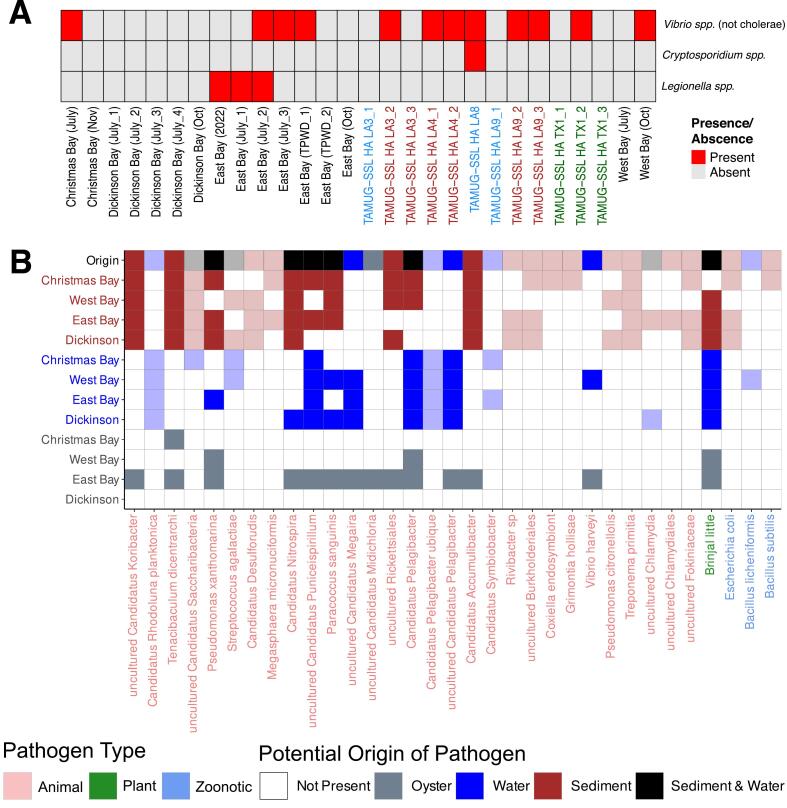
A) Pathogens detected using the BF-GI and LyoKit in red. Only 3 of the 22 pathogens tested for were identified. Column names correspond to the samples in Supp. Table 2 and the color of the text corresponds to post-harvest treatments, individual quick freezing (blue), high hydrostatic pressure (red), and gamma irradiation (green), and no treatment (black). B) Pathogens identified by comparing 16S rRNA gene sequences to the MBPD database [35]. Column names are colored according to pathogen type in MBPD. Colors of boxes represent the sample type, and in the top row, a summarized representation of non-oyster presence is used. Lightly colored columns are pathogens that were not detected in oysters but were detected in the water or sediment. (For interpretation of the references to colour in this figure legend, the reader is referred to the web version of this article.)

We tested 28 individual oysters for human pathogens using the BF-GI. No human pathogen was detected in 14 of the 28 oyster slurries tested (Fig. 5A). *Vibrio* spp. but not *V. cholerae,* was detected in 12 oysters positive for human pathogens (Fig. 5A). Six of the oysters positive for *Vibrio* spp. were collected in Galveston Bay: four from East Bay including one treated with gamma irradiation, one from Christmas Bay, and one from West Bay (Fig. 5 A, Supp. Table 2). The remaining six *Vibrio* spp. positive oysters were from Louisiana commercial oyster farms, with five pre-treated using high hydrostatic pressure and one with quick freezing, which also tested positive for *Cryptosporidium* (Fig. 5A). Low levels of *Legionella* spp.*,* but not L. *pneumophila* or L. *pneumophila* serogroup 1, were detected in three samples, all from East Bay, however, several samples had PCR inhibition as determined by the absence of internal control (Fig. 5A, Supp. Table 2).

We identified potential animal, plant, and zoonotic pathogenic bacteria using the MBPD database. All oysters tested contained potential animal and plant pathogens (Fig. 5B). Of the 33 pathogens identified, 14 (42 %) were found in oyster tissues (Fig. 5B). Of the 14 pathogens found in oyster tissues, all were identified in East Bay oysters; one in Christmas Bay, and three in the West Bay oysters (Fig. 5B). Furthermore, 71 % and 57 % of the pathogens detected in the oysters were also found in the sediment and water column, respectively (Fig. 5B).

## 4 Discussion

### 4.1 The potential for oyster microbiomes and viromes as sentinels of human and ecosystem health

Most of the viral genes identified in this study were unique to the samples, with known viral genes largely identified as the cosmopolitan *Myoviridae*, *Podoviridae*, and *Siphoviridae,* in agreement with previous investigations of the viromes of oysters [10,18]. In contrast to previous studies, we found that the next most abundant families were *Phycodnaviridae* and *Mimiviridae,* which are known to infect marine algal species. Oysters more efficiently filter particles >3 μm but are known to also filter particles <1 μm which would include these giant viruses; however, they have been shown to have little impact on the quantity of viral-like particles in the water column [40,41]. We identified viral families, *Metaviridae* and *Poxviridae*, that infect a variety of eukaryotes including humans, however, the BF-GI was not able to detect common, known viral pathogens of humans.

The striking similarities between viromes of the environmental samples and the oysters imply that the specific viral families found in oysters are not enriched as was seen in the bacterial and eukaryotic populations, suggesting that most viruses found in oysters are transient. This similarity between viruses in the oysters and the surrounding environment offers the potential for assessing the health of the ecosystem by exploring the viral communities of oysters being harvested within an ecosystem. Our analysis of the gene functions present within oyster viromes shows potential for using oysters as indicators of ecosystem function. Additional research into characterizing the unidentifiable viral sequences and tracking known viruses that infect humans will help to resolve the ability to use oyster viromes as sentinels of both human and ecosystem health.

The Mycoplasmatales, Fusobacteriales, and Spirochaetales bacterial orders have been found in higher abundance in oysters and have been previously described as core constituents of the oyster microbiome [14,[42], [43], [44], [45]]. *Mycoplasma* has been associated with the spread of disease and subsequent mortality in oysters [46,47], as well as associated with healthier oysters [43,48], highlighting its opportunistic pathogenicity. Similarly, Spirochaetales and Fusobacteriales are widely distributed taxa often associated with gut microbiomes and marine environments; they both include members considered to be opportunistic pathogens. The NMDS analysis showed that the East Bay oyster communities were most different from those of Christmas and West Bay with reductions in these core oyster microbiome constituents and increases in Synechococcales. Taken with the higher incidence of *Vibrio* spp. and *Legionella* spp. detected in East Bay via MDPB, LyoKit, and BF-GI, this suggests that changes in the core bacterial communities of oysters could reflect increased risk of human pathogens. Future studies using increased sample sizes and in vitro manipulation of oysters could elucidate connections between changes in these key microbial groups and overall oyster health and the incidence of pathogens.

### 4.2 Adapting oyster farming, harvesting, and treatment with microbes in mind

As the aquaculture of bivalves, including oysters, is expanding [9], it is imperative to understand the factors affecting pathogen incidence in commercial farms. This is of particular concern in Texas estuaries with the recent (2018) legalization and permitting of oyster mariculture operations [49].While the benefits of oyster aquaculture are diverse [5], the identification of pathogens using the MBPD database suggests that many potential pathogens associated with oysters can be found in the surrounding sediment. Whether pathogens are sourced from the sediment or deposited in the form of biodeposits from the oysters, the sediment represents a potential reservoir for pathogens to be introduced, or even reintroduced, to oysters [50]. This could have implications in determining the best farming methods to use, i.e. off-bottom methods like hanging cages could reduce contact between oysters and sediments. Additionally, the presence of *Legionella* spp. in oysters warrants further assessment of the proximity of oyster farms to freshwater outflows. Indeed, *Legionella* spp. is normally found in freshwater or anthropogenically influenced run-off and can survive in marine habitats [[51], [52], [53]].

Current oyster pathogen control measures focus on monitoring efforts and post-harvest processing of harvested oysters [54]. Monitoring efforts generally involve culturing of water quality indicator species such as *E. coli* and *Enterococcus, Vibrio parahaemolyticus* and toxic algal species [54]. To mitigate the risks of pathogens by reducing bacterial counts, post-harvest treatments include thermal processing, freezing, irradiation, or high pressure [54]. However, these methods are not always able to eliminate pathogens [19,20]. We identified *Vibrio* spp. in 39 % of the oysters tested, including seven in which post-harvest processing was conducted. Additionally, using the BF-GI and LyoKit we identified oysters positive for *Cryptosporidium* and *Legionella* spp.*,* which are human pathogens not included in typical monitoring efforts [54]. Our results suggest that current monitoring and treatment techniques used by regulatory agencies are likely failing to identify and/or mitigate potential pathogens, whether introduced in the environment or during harvesting. Additional studies should be done to confirm the infectivity of the pathogens detected using these techniques.

The results of the MBPD database analysis suggest that there are more pathogens present than the BioFire system detected. Further research should be conducted to confirm the reliability of the molecular techniques used, including positive and negative oyster slurry control and additional negative controls throughout the sampling process. Additionally, future studies could be improved by increasing the number of samples, the use of replicates for each site, sampling over time to address seasonal differences, assessing the health of oysters collected, and the use of additional pathogen-specific PCR primers. The improvement and expansion of the set of primers to identify human pathogens used by the BF-GI would be of particular interest to public health and regulatory agencies to provide seafood safety laboratories that are largely dependent on culture-based techniques. Molecular techniques can provide benefits over culturing such as decreased turn-around times, increased sensitivity, ability to identify multiple pathogens including protists and viruses, and enhanced ability to identify pathogens that are unculturable.

## 5 Conclusions

Our results highlight the potential novel use of molecular tools to detect pathogens that may be missed in routine screening. We showed that methods being used in the healthcare system, such as the BioFire FilmArray, could be adapted to provide high-throughput detection of pathogens in food safety programs. Additionally, our research showed how the bacterial communities of oysters are unique and largely made up of core constituents not abundant in the surrounding water or sediment. On the other hand, the viral communities within oysters largely mirror the composition and function of the surrounding water. Finally, we suggest that while monitoring of bacterial communities of oysters provides an avenue to assess health risks to humans, analysis of oyster viral communities may have the potential to assess ecosystem health due to their high level of similarity.

## CRediT authorship contribution statement

**Jordan R. Walker:** Writing – original draft, Visualization, Software, Methodology, Investigation, Formal analysis, Conceptualization. **Dennis A. Bente:** Writing – review & editing, Supervision, Project administration, Funding acquisition. **Megan T. Burch:** Writing – review & editing, Validation, Investigation. **Filipe M. Cerqueira:** Methodology, Investigation. **Ping Ren:** Investigation, Methodology. **Jessica M. Labonté:** Writing – review & editing, Supervision, Project administration, Funding acquisition.

## Declaration of competing interest

The authors declare that they have no known competing financial interests or personal relationships that could have appeared to influence the work reported in this paper.

## Data Availability

Quality controlled sequence data will be made available via the NCBI Short Read Archive (SRA) pending acceptance, under BioProject PRJNA1166149.

## Appendix A Supplementary data

Supplementary material Image 1
