# Supplementary material for: Molecular assessment of oyster microbiomes and viromes reveals their potential as pathogen and ecological sentinels
Source: One Health. 2025 Jan 13;20:100973. doi: 10.1016/j.onehlt.2025.100973 (PMC11786891; doi:10.1016/j.onehlt.2025.100973)
Supplement: Supplementary file 1 — Supplementary material [file mmc1.docx]

**Supplementary Table 1.** Sample locations and abiotoc conditions for the samples that were used for metagenomic, 16S rRNA, and virome generation.

| **Region** | **Longitude/**  **Latitude** | **Date**  **Collected** | **Depth (m)** | **Temperature (∞C)** | **Salinity (ppt)** | **DO (ml L-1)** |
| --- | --- | --- | --- | --- | --- | --- |
| Dickinson Bay | 29.48N 94.89W | 10/12/23 | 2.34 | 22.13 | 26.87 | 7.18 |
| East Bay | 29.48N 94.77W | 10/17/23 | 1.91 | 18.35 | 26.9 | 8.6 |
| West Bay | 29.26N 94.91W | 10/24/23 | 1.88 | 25.23 | 30.57 | 6.49 |
| Christmas Bay | 28.99N 95.23W | 11/2/23 | 0.3 | 13.7 | 32.8 | 7.3 |

**Supplementary Table 2.** Sample locations used for pathogen detection with the adapted BioFire ® Film Array ® protocol. Sample starting with HA were collected from the Texas A&M University at Galveston Seafood Safety Lab (TAMUG-SSL). The HA samples were processed on August 1, 2023, at Texas A&M University at Galveston. The HA locations correspond to harvesting areas as defined by Texas Health and Human Services and Louisiana Department of Health. Oysters obtained from the TAMUG-SSL were treated by either gamma irradiation (GI), high hydrostatic pressure (HHP), and individual quick freezing (IQF).

| **Location** | **Sample Date** | **N Oysters** | **Treatment Type** | **Treatment Date** | **Biofire Detection** | ***Legionella spp.*** | **Collected From** |
| --- | --- | --- | --- | --- | --- | --- | --- |
| Christmas Bay-7 | 7/19/23 | 5 | NA | NA | *Vibrio spp.* (not *cholerae*) | - | Environment |
| Christmas Bay-11 | 11/2/23 | 7 | NA | NA | none | - | Environment |
| Dickinson Bay-7_1 | 7/17/23 | 10 | NA | NA | none | - | Environment |
| Dickinson Bay-7_2 | 7/17/23 | 10 | NA | NA | none | - | Environment |
| Dickinson Bay-7_3 | 7/17/23 | 10 | NA | NA | none | - | Environment |
| Dickinson Bay-7_4 | 7/17/23 | 10 | NA | NA | none | - | Environment |
| Dickinson Bay-10 | 10/12/23 | 10 | NA | NA | none | - | Environment |
| East Bay-22 | 10/3/22 | 2 | NA | NA | none | + | TAMUG-Archived |
| East Bay-7 | 7/17/23 | 10 | NA | NA | none | + | Environment |
| East Bay-7_1 | 7/17/23 | 10 | NA | NA | *Vibrio spp.* (not *cholerae*) | + | Environment |
| East Bay-7_2 | 7/17/23 | 10 | NA | NA | *Vibrio spp.* (not *cholerae*) | - | Environment |
| East Bay-TPWD_1 | 7/20/23 | 6 | NA | NA | *Vibrio spp.* (not *cholerae*) | - | TPWD |
| East Bay-TPWD_2 | 7/20/23 | 6 | NA | NA | none | - | TPWD |
| East Bay-10 | 10/17/23 | 7 | NA | NA | none | - | Environment |
| HA LA3_1 | 6/20/23 | 12 | IQF | 6/21/23 | none | - | TAMUG-SSL |
| HA LA3_2 | 7/28/23 | 12 | HHP | 7/31/23 | *Vibrio spp.* (not *cholerae*) | - | TAMUG-SSL |
| HA LA3_3 | 7/28/23 | 12 | HHP | 7/31/23 | none | - | TAMUG-SSL |
| HA LA4_1 | 7/28/23 | 12 | HHP | 7/31/23 | *Vibrio spp.* (not *cholerae*) | - | TAMUG-SSL |
| HA LA4_2 | 7/28/23 | 12 | HHP | 7/31/23 | *Vibrio spp.* (not *cholerae*) | - | TAMUG-SSL |
| HA LA8 | 6/7/23 | 12 | IQF | 6/12/23 | *Vibrio spp.* (not *cholerae*), *Cryptosporidium* | - | TAMUG-SSL |
| HA LA9_1 | 6/9/23 | 12 | IQF | 6/12/23 | none | - | TAMUG-SSL |
| HA LA9_2 | 7/27/23 | 12 | HHP | 7/31/23 | *Vibrio spp.* (not *cholerae*) | - | TAMUG-SSL |
| HA LA9_3 | 7/27/23 | 12 | HHP | 7/31/23 | *Vibrio spp.* (not *cholerae*) | - | TAMUG-SSL |
| HA TX1_1 | 7/29/23 | 12 | GI | 7/31/23 | none | - | TAMUG-SSL |
| HA TX1_2 | 7/29/23 | 12 | GI | 7/31/23 | *Vibrio spp.* (not *cholerae*) | - | TAMUG-SSL |
| HA TX1_3 | 7/29/23 | 12 | GI | 7/31/23 | none | - | TAMUG-SSL |
| West Bay-7 | 7/19/23 | 6 | NA | NA | none | - | Environment |
| West Bay-10 | 10/24/23 | 10 | NA | NA | *Vibrio spp.* (not *cholerae*) | - | Environment |

**Supplementary Table 3.** Pathogens detected with the adapted BioFire ® Film Array ® protocol.

| **Pathogen Type** | **Genus** | **Species** |
| --- | --- | --- |
| Bacteria | *Campylobacter* | *C. jejuni* |
|  |  | *C. coli* |
|  |  | *C. upsaliensis* |
|  | *Clostridioides* | *C. difficile* (toxin A/B) |
|  | *Plesiomonas* | *P. shigelloides* |
|  | *Salmonella* | *S. spp.* |
|  | Yersinia | *Y. enterocolitica* |
|  | *Vibrio* | *V. parahaemolyticus* |
|  |  | *V. vulnificus* |
|  |  | *V. cholerae* |
|  |  | *V. spp.* |
|  | *Escherichia/Shigella* | Enteroaggregative *E. coli* (EAEC) |
|  |  | Enteropathogenic *E. coli* (EPEC) |
|  |  | Enterotoxigenic *E. coli* (ETEC) lt/st |
|  |  | Shiga-like toxin-producing *E. coli* (STEC) stx1/stx2 |
|  |  | *E. coli* O157 |
|  |  | *Shigella*/Enteroinvasive *E. coli* (EIEC) |
| Parasites | *Cryptosporidium* | *C. spp.* |
|  | *Cyclospora* | *C. cayetanensis* |
|  | *Entamoeba* | *E. histolytica* |
|  | *Giardia* | *G. lamblia* |
| Viruses |  | Adenovirus F40/41 |
|  |  | Astrovirus |
|  |  | Norovirus GI/GII |
|  |  | Rotavirus A |
|  |  | Sapovirus (I, II, IV, and V) |

**Supplementary Table 4.** Quality control and assembly statistics of the metagenomes generated.

| **Sample** | **Pairs** | **Joined** | **Merged/ Unmerged** | **Reads**  **After Art. Trim** | **Reads %** | **Reads**  **After Adapt. Trim** | **Read%** | **Contam**  **reads** | **Contam reads %** | **Entropy Masking (bases)** | **Final reads** | **% Read Left** | **Final bases** | **% Bases Left** | **Contigs Assembled** | **Average Length** | **N50** |
| --- | --- | --- | --- | --- | --- | --- | --- | --- | --- | --- | --- | --- | --- | --- | --- | --- | --- |
| DBO_V_L001 | 39,764,595 | 14,617,870 | merged | 14,617,824 | 100% | 14,617,690 | 100% | 174 | 0% | 88,450 | 14,617,516 | 100% | 3,567,944,136 | 100% | 329,565 | 2,197 | 2,321 |
|  |  |  | unmerged | 50,293,228 | 100% | 50,068,256 | 100% | 89,873 | 0% | 8,459,169 | 49,895,332 | 99% | 7,071,818,546 | 94% |  |  |  |
| DBO_V_L002 | 40,953,903 | 15,113,983 | merged | 15,113,938 | 100% | 15,113,794 | 100% | 184 | 0% | 94,417 | 15,113,610 | 100% | 3,700,744,879 | 100% |  |  |  |
|  |  |  | unmerged | 51,679,572 | 100% | 51,480,048 | 100% | 68,594 | 0% | 7,987,626 | 51,350,812 | 99% | 7,401,306,529 | 95% |  |  |  |
| DBS_T_L001 | 22,334,762 | 8,066,143 | merged | 8,066,122 | 100% | 8,066,061 | 100% | 227 | 0% | 56,803 | 8,065,834 | 100% | 1,938,147,623 | 100% | 245,628 | 2,216 | 2,338 |
|  |  |  | unmerged | 28,537,166 | 100% | 28,441,916 | 100% | 56,675 | 0% | 3,778,535 | 28,333,994 | 99% | 3,985,898,456 | 93% |  |  |  |
| DBS_T_L002 | 22,793,215 | 8,316,891 | merged | 8,316,871 | 100% | 8,316,814 | 100% | 200 | 0% | 62,247 | 8,316,614 | 100% | 2,005,812,184 | 100% |  |  |  |
|  |  |  | unmerged | 28,952,574 | 100% | 28,877,776 | 100% | 41,535 | 0% | 3,432,476 | 28,800,082 | 99% | 4,130,457,853 | 95% |  |  |  |
| DBW_B_L001 | 28,515,004 | 11,701,954 | merged | 11,701,901 | 100% | 11,701,800 | 100% | 90 | 0% | 91,043 | 11,701,710 | 100% | 2,681,913,138 | 100% | 287,459 | 2,231 | 2,399 |
|  |  |  | unmerged | 33,625,922 | 100% | 33,482,116 | 100% | 68,056 | 0% | 6,581,391 | 33,352,364 | 99% | 4,689,116,591 | 93% |  |  |  |
| DBW_B_L002 | 29,121,414 | 12,078,382 | merged | 12,078,329 | 100% | 12,078,220 | 100% | 97 | 0% | 91,512 | 12,078,123 | 100% | 2,777,539,164 | 100% |  |  |  |
|  |  |  | unmerged | 34,085,926 | 100% | 33,966,012 | 100% | 50,126 | 0% | 5,757,438 | 33,872,328 | 99% | 4,855,624,460 | 95% |  |  |  |
| DBW_V_L001 | 83,029,689 | 28,689,766 | merged | 28,689,600 | 100% | 28,689,510 | 100% | 626 | 0% | 207,893 | 28,688,884 | 100% | 6,972,988,920 | 100% | 556,009 | 2,233 | 2,373 |
|  |  |  | unmerged | 108,679,228 | 100% | 108,289,560 | 100% | 221,064 | 0% | 9,874,339 | 107,861,862 | 99% | 15,190,064,952 | 93% |  |  |  |
| DBW_V_L002 | 84,868,756 | 29,465,641 | merged | 29,465,507 | 100% | 29,465,412 | 100% | 664 | 0% | 213,912 | 29,464,748 | 100% | 7,183,389,006 | 100% |  |  |  |
|  |  |  | unmerged | 110,805,590 | 100% | 110,487,296 | 100% | 164,714 | 0% | 8,447,245 | 110,177,134 | 99% | 15,812,139,943 | 95% |  |  |  |
| EBO_V_L001 | 38,104,892 | 17,665,949 | merged | 17,665,947 | 100% | 17,665,390 | 100% | 170 | 0% | 100,408 | 17,665,220 | 100% | 3,774,941,966 | 100% | 155,931 | 1,864 | 1,791 |
|  |  |  | unmerged | 40,877,886 | 100% | 40,568,268 | 99% | 61,061 | 0% | 6,944,940 | 40,453,040 | 99% | 5,690,153,181 | 93% |  |  |  |
| EBO_V_L002 | 39,293,676 | 18,358,416 | merged | 18,358,415 | 100% | 18,357,871 | 100% | 172 | 0% | 115,837 | 18,357,699 | 100% | 3,934,773,992 | 100% |  |  |  |
|  |  |  | unmerged | 41,870,520 | 100% | 41,582,814 | 99% | 48,323 | 0% | 6,576,591 | 41,493,202 | 99% | 5,940,025,495 | 95% |  |  |  |
| EBS_T_L001 | 29,297,370 | 12,754,067 | merged | 12,754,065 | 100% | 12,753,867 | 100% | 31 | 0% | 39,215 | 12,753,836 | 100% | 2,752,220,836 | 100% | 140,762 | 1,937 | 1,875 |
|  |  |  | unmerged | 33,086,592 | 100% | 32,933,012 | 100% | 50,469 | 0% | 3,730,707 | 32,836,992 | 99% | 4,614,143,526 | 93% |  |  |  |
| EBS_T_L002 | 30,052,266 | 13,218,823 | merged | 13,218,822 | 100% | 13,218,588 | 100% | 26 | 0% | 39,902 | 13,218,562 | 100% | 2,860,116,257 | 100% |  |  |  |
|  |  |  | unmerged | 33,666,870 | 100% | 33,536,206 | 100% | 38,489 | 0% | 3,217,667 | 33,464,088 | 99% | 4,791,281,287 | 95% |  |  |  |
| EBW_B_L001 | 27,951,967 | 8,334,964 | merged | 8,334,964 | 100% | 8,334,906 | 100% | 11 | 0% | 173,611 | 8,334,895 | 100% | 1,847,858,069 | 100% | 166,111 | 1,888 | 1,811 |
|  |  |  | unmerged | 39,234,002 | 100% | 38,998,416 | 99% | 70,061 | 0% | 8,298,906 | 38,868,852 | 99% | 5,442,446,888 | 92% |  |  |  |
| EBW_B_L002 | 28,908,431 | 8,644,269 | merged | 8,644,269 | 100% | 8,644,185 | 100% | 11 | 0% | 186,420 | 8,644,174 | 100% | 1,922,605,374 | 100% |  |  |  |
|  |  |  | unmerged | 40,528,322 | 100% | 40,321,744 | 99% | 57,034 | 0% | 7,863,116 | 40,217,930 | 99% | 5,732,033,680 | 94% |  |  |  |
| EBW_V_L001 | 33,520,376 | 9,062,431 | merged | 9,062,431 | 100% | 9,062,308 | 100% | 6 | 0% | 107,017 | 9,062,302 | 100% | 2,006,530,606 | 100% | 200,359 | 1,812 | 1,760 |
|  |  |  | unmerged | 48,915,882 | 100% | 48,704,820 | 100% | 73,491 | 0% | 5,637,905 | 48,564,202 | 99% | 6,831,347,745 | 93% |  |  |  |
| EBW_V_L002 | 34,847,230 | 9,434,352 | merged | 9,434,352 | 100% | 9,434,244 | 100% | 6 | 0% | 115,702 | 9,434,238 | 100% | 2,095,319,868 | 100% |  |  |  |
|  |  |  | unmerged | 50,825,752 | 100% | 50,658,712 | 100% | 59,497 | 0% | 4,843,451 | 50,546,562 | 99% | 7,234,291,976 | 95% |  |  |  |
| WBO_V_L001 | 33,556,725 | 19,447,935 | merged | 19,447,935 | 100% | 19,447,342 | 100% | 266 | 0% | 1,441,124 | 19,447,076 | 100% | 2,193,833,902 | 100% | 14,748 | 1,353 | 1,282 |
|  |  |  | unmerged | 28,217,572 | 100% | 26,849,156 | 95% | 583,670 | 4% | 237,749,188 | 26,109,166 | 93% | 1,663,719,137 | 39% |  |  |  |
| WBO_V_L002 | 34,567,113 | 19,181,872 | merged | 19,181,872 | 100% | 19,181,161 | 100% | 212 | 0% | 1,679,526 | 19,180,949 | 100% | 2,211,789,325 | 100% |  |  |  |
|  |  |  | unmerged | 30,770,468 | 100% | 29,321,238 | 95% | 594,639 | 4% | 252,311,197 | 28,577,394 | 93% | 1,805,641,196 | 39% |  |  |  |
| WBS_T_L001 | 3,332,862 | 1,210,116 | merged | 1,210,116 | 100% | 1,210,057 | 100% | 82 | 0% | 25,197 | 1,209,975 | 100% | 265,459,797 | 99% | 9,105 | 1,988 | 2,015 |
|  |  |  | unmerged | 4,245,492 | 100% | 3,637,948 | 86% | 9,243 | 1% | 1,023,376 | 3,620,428 | 85% | 497,262,159 | 78% |  |  |  |
| WBS_T_L002 | 3,427,185 | 1,246,517 | merged | 1,246,517 | 100% | 1,246,468 | 100% | 66 | 0% | 26,620 | 1,246,402 | 100% | 274,298,475 | 100% |  |  |  |
|  |  |  | unmerged | 4,361,336 | 100% | 3,714,304 | 85% | 7,274 | 0% | 927,019 | 3,700,718 | 85% | 518,777,205 | 79% |  |  |  |
| WBW_B_L001 | 38,415,961 | 23,281,578 | merged | 23,281,578 | 100% | 23,281,029 | 100% | 531 | 0% | 772,698 | 23,280,498 | 100% | 4,526,199,385 | 100% | 138,179 | 1,754 | 1,721 |
|  |  |  | unmerged | 30,268,754 | 100% | 26,519,672 | 88% | 141,069 | 1% | 20,084,408 | 26,279,364 | 87% | 3,155,370,672 | 69% |  |  |  |
| WBW_B_L002 | 39,168,854 | 23,659,429 | merged | 23,659,429 | 100% | 23,658,896 | 100% | 509 | 0% | 895,037 | 23,658,387 | 100% | 4,638,879,744 | 100% |  |  |  |
|  |  |  | unmerged | 31,018,844 | 100% | 26,985,618 | 87% | 126,693 | 1% | 19,294,504 | 26,775,622 | 86% | 3,228,896,063 | 69% |  |  |  |
| WBW_V_L001 | 41,935,070 | 24,372,001 | merged | 24,372,001 | 100% | 24,371,349 | 100% | 283 | 0% | 2,638,921 | 24,371,066 | 100% | 3,357,751,639 | 100% | 21,711 | 1,426 | 1,338 |
|  |  |  | unmerged | 35,126,138 | 100% | 34,510,972 | 98% | 486,429 | 3% | 73,809,343 | 33,816,684 | 96% | 2,946,962,061 | 56% |  |  |  |
| WBW_V_L002 | 43,123,066 | 24,300,978 | merged | 24,300,978 | 100% | 24,300,237 | 100% | 193 | 0% | 2,976,563 | 24,300,044 | 100% | 3,416,727,521 | 100% |  |  |  |
|  |  |  | unmerged | 37,644,176 | 100% | 37,009,628 | 98% | 487,022 | 3% | 76,662,448 | 36,327,428 | 97% | 3,143,814,695 | 56% |  |  |  |
| XBO_V_L001 | 18,140,712 | 5,019,260 | merged | 5,019,260 | 100% | 5,019,177 | 100% | 514 | 0% | 10,966 | 5,018,663 | 100% | 1,267,301,037 | 99% | 167,117 | 2,225 | 2,321 |
|  |  |  | unmerged | 26,242,880 | 100% | 26,114,142 | 100% | 54,903 | 0% | 3,402,580 | 26,007,100 | 99% | 3,624,230,983 | 92% |  |  |  |
| XBO_V_L002 | 18,625,940 | 5,176,260 | merged | 5,176,260 | 100% | 5,176,159 | 100% | 495 | 0% | 13,349 | 5,175,664 | 100% | 1,312,785,641 | 99% |  |  |  |
|  |  |  | unmerged | 26,899,352 | 100% | 26,794,352 | 100% | 39,493 | 0% | 2,692,892 | 26,718,376 | 99% | 3,799,608,733 | 94% |  |  |  |
| XBS_T_L001 | 18,332,761 | 5,989,130 | merged | 5,989,130 | 100% | 5,988,856 | 100% | 27 | 0% | 14,978 | 5,988,829 | 100% | 1,524,477,395 | 99% | 153,167 | 2,266 | 2,454 |
|  |  |  | unmerged | 24,687,260 | 100% | 24,547,924 | 99% | 42,532 | 0% | 5,356,635 | 24,464,780 | 99% | 3,451,224,115 | 93% |  |  |  |
| XBS_T_L002 | 18,727,185 | 6,157,481 | merged | 6,157,481 | 100% | 6,157,142 | 100% | 39 | 0% | 13,675 | 6,157,103 | 100% | 1,572,544,530 | 100% |  |  |  |
|  |  |  | unmerged | 25,139,404 | 100% | 25,017,100 | 100% | 29,640 | 0% | 4,531,982 | 24,959,674 | 99% | 3,589,286,544 | 95% |  |  |  |
| XBW_B_L001 | 27,539,764 | 14,857,322 | merged | 14,857,309 | 100% | 14,856,904 | 100% | 101 | 0% | 18,840 | 14,856,803 | 100% | 3,394,926,808 | 100% | 153,270 | 2,315 | 2,507 |
|  |  |  | unmerged | 25,364,872 | 100% | 25,158,824 | 99% | 50,115 | 0% | 7,174,053 | 25,060,928 | 99% | 3,496,848,181 | 92% |  |  |  |
| XBW_B_L002 | 27,900,891 | 15,206,841 | merged | 15,206,833 | 100% | 15,206,365 | 100% | 99 | 0% | 20,354 | 15,206,266 | 100% | 3,485,880,444 | 100% |  |  |  |
|  |  |  | unmerged | 25,388,086 | 100% | 25,198,866 | 99% | 34,463 | 0% | 5,570,486 | 25,132,190 | 99% | 3,585,337,713 | 94% |  |  |  |
| XBW_V_L001 | 22,432,888 | 9,603,849 | merged | 9,603,840 | 100% | 9,603,497 | 100% | 81 | 0% | 37,372 | 9,603,416 | 100% | 2,137,854,070 | 100% | 138,055 | 2,086 | 2,131 |
|  | 22,980,527 | 9,864,599 | unmerged | 25,658,062 | 100% | 25,493,464 | 99% | 42,248 | 0% | 5,455,956 | 25,411,010 | 99% | 3,592,718,880 | 93% |  |  |  |
| XBW_V_L002 | 22,980,527 | 9,864,599 | merged | 9,864,594 | 100% | 9,864,278 | 100% | 94 | 0% | 31,739 | 9,864,184 | 100% | 2,201,460,117 | 100% |  |  |  |
|  |  |  | unmerged | 26,231,844 | 100% | 26,075,942 | 99% | 31,263 | 0% | 4,988,747 | 26,015,568 | 99% | 3,743,387,545 | 95% |  |  |  |
